# Clinical-Community Partnerships to Identify Patients With Food Insecurity and Address Food Needs

**DOI:** 10.5888/pcd14.170343

**Published:** 2017-11-16

**Authors:** Elizabeth A. Lundeen, Karen R. Siegel, Holly Calhoun, Sonia A. Kim, Sandra P. Garcia, Natalie M. Hoeting, Diane M. Harris, Laura Kettel Khan, Bryce Smith, Heidi M. Blanck, Kevin Barnett, Anne C. Haddix

**Affiliations:** 1 Centers for Disease Control and Prevention, Atlanta, Georgia; 2 Public Health Institute, Oakland, California; 3 Alliance for a Healthier Generation, New York, New York; 4 Emory University School of Medicine, Atlanta, Georgia; 5 CDC Foundation, Atlanta, Georgia

## Abstract

**Introduction:**

More than 42 million people in the United States are food insecure. Although some health care entities are addressing food insecurity among patients because of associations with disease risk and management, little is known about the components of these initiatives.

**Methods:**

The Systematic Screening and Assessment Method was used to conduct a landscape assessment of US health care entity–based programs that screen patients for food insecurity and connect them with food resources. A network of food insecurity researchers, experts, and practitioners identified 57 programs, 22 of which met the inclusion criteria of being health care entities that 1) screen patients for food insecurity, 2) link patients to food resources, and 3) target patients including adults aged 50 years or older (a focus of this assessment). Data on key features of each program were abstracted from documentation and telephone interviews.

**Results:**

Most programs (n = 13) focus on patients with chronic disease, and most (n = 12) partner with food banks. Common interventions include referrals to or a list of food resources (n = 19), case managers who navigate patients to resources (n = 15), assistance with federal benefit applications (n = 14), patient education and skill building (n = 13), and distribution of fruit and vegetable vouchers redeemable at farmers markets (n = 8). Most programs (n = 14) routinely screen all patients.

**Conclusion:**

The programs reviewed use various strategies to screen patients, including older adults, for food insecurity and to connect them to food resources. Research is needed on program effectiveness in improving patient outcomes. Such evidence can be used to inform the investments of potential stakeholders, including health care entities, community organizations, and insurers.

## Introduction

Nearly 13% of US households — 42.2 million people — were food insecure at some time during 2015 (1,2). The US Department of Agriculture (USDA) defines food insecurity as an economic and social condition characterized by limited or uncertain access to adequate food (3). Together with insecurity in housing and income, food insecurity is a social issue that affects health. Food insecurity is associated with a higher risk of chronic disease, including obesity (4–6), diabetes (4,7–9), depression (4), hypertension (7,10), and chronic kidney disease (11). Among individuals with diabetes, food insecurity is associated with poorer glycemic control (12–15) and greater health care use, including outpatient visits (13), emergency department visits (16) and hospitalizations (17).

As a result of the growing body of evidence linking food insecurity and health, health care entities, including hospitals, health systems, and clinics, are increasingly attempting to address food insecurity in the communities they serve. Some payment systems are shifting from traditional fee-for-service to value-based reimbursement, which has incented many hospitals to consider community factors and social issues that affect health, with a goal of reducing the demand for treatment of preventable conditions. Additionally, expansion of health insurance coverage models resulted for many health systems in a reduced need for provision of charitable care in nonprofit hospitals, and therefore increased the need to find other community benefit investments to maintain a tax-exempt status.

Although anecdotal evidence suggests a growing role of hospitals and health care systems in addressing food insecurity, a description of the range of programs being implemented in the United States is lacking. This article reports on the results of a landscape assessment of health care entity–based programs in the United States that screen patients, especially older adults, for food insecurity and then connect them with food resources.

## Methods

As part of the Tackling Hunger to Improve Health in Americans project (Tackling Hunger) (18), from January through August of 2016, the Public Health Institute (PHI) conducted the first phase of an evaluability assessment, to identify US health care entity–based programs that screen patients for food insecurity and connect them with food resources. The methodology for this evaluability assessment was based on the Systematic Screening and Assessment Method, which identifies interventions and determines their potential for formal evaluation (19). The Centers for Disease Control and Prevention (CDC), which provided technical assistance during the initial phase of this project, reviewed the data collected to describe the landscape of health care entity–based programs engaged in addressing food insecurity.

The first step in the evaluability assessment was to identify relevant programs through a nomination process conducted by professionals and experts in the field, an internet search for reports and white papers, and a literature review in PubMed. There were 3 inclusion criteria for programs to be selected for this assessment: 1) health care entity conducts screening to identify patients with food insecurity through the use of assessment tools, 2) health care entity links the patient to food security resources or programs, and 3) target patient population includes adults aged 50 years or older. Health care entity–based programs that focus on seniors (aged ≥50 y) with chronic disease were identified as a population of interest, because of priorities of the project funders. An online nomination request form was distributed widely through the e-newsletters and electronic mailing lists of various professional networks involved in the areas of health care and food insecurity and was also sent to a targeted group of professionals working in this area; programs identified through the internet search and literature review were also sent the nomination form and invited to self-nominate.

Self-nominations and third-party nominations were accepted from March through June 2016, yielding 57 programs. Managers of nominated programs were then contacted to request additional information to verify whether the program met the criteria. The project team then reviewed the nomination forms and additional documentation. Three staff members were assigned to review each program and independently determine whether the program met each of 3 inclusion criteria. If the 3 reviewers did not independently reach the same conclusion for inclusion criteria, they discussed the program; if consensus among the 3 reviewers was not achieved, a fourth member of the team reviewed the program. Of the 57 nominated programs, 22 met all 3 inclusion criteria.

A telephone interview was conducted with each of the program managers in August 2016. The purpose of the interview was to gather additional information and to clarify information the program had previously provided, including program objectives, activities, target population and reach, the role of the hospital or health care system in food insecurity screening and program implementation, food insecurity screening tools and processes, program stakeholders and partners, funding, sustainability, data collection, and program evaluation activities.

This landscape assessment presents data on key features of the 22 health care entity–based programs; program names were blinded and identified by using letters of the alphabet. The programs featured in this landscape assessment are geographically diverse, with 7 programs from the northeast region, 5 from the western region, 4 from the midwestern region, 3 from the southwestern region, 1 from the mountain plains region, 1 from the mid-Atlantic region, and 1 from the southeast region (20). Most programs started between 2014 and 2016, and all but 3 of the programs began after 2010.

## Results

The programs are implemented by nonprofit health care entities, and program implementation occurs in various settings, including nonprofit hospitals and health systems, hospital-affiliated primary care clinics, academic health centers, teaching hospitals, community hospitals, critical-access hospitals, acute-care hospitals, children’s hospitals, federally qualified health centers (FQHCs), mental health and substance abuse treatment centers, safety-net clinics and hospitals, county- or state-run health centers, large integrated care delivery health systems, and nonprofit dental clinics (Table 1). Most programs (n = 18) target patients of all ages with food insecurity; however, several programs indicated a focus on adults aged 50 years or older (n = 7), and nearly half (n = 10) mentioned a special interest in addressing food insecurity among children while still capturing adults aged 50 years or older through screening and intervention. Most programs (n = 13) focus on addressing the needs of patients with food insecurity and chronic disease. The programs are most often led by the health care entity, and most patients being served by the programs are uninsured or insured through Medicaid or Medicare. More than half of the programs involve collaborations with a local or regional food bank, the most commonly reported program partner. However, a range of partners participate in program implementation, including local and regional food banks, universities and schools of medicine, city and county health departments, local health councils, farms, farmers markets, food and agriculture networks, social service agencies, schools, hunger networks, senior centers, and community nonprofit organizations focusing on health, food insecurity, seniors, and early childhood.

**Table 1:** Characteristics of 22 Programs Identified as Health Care Entity–Based Food Insecurity Interventions, Tackling Hunger Landscape Assessment, United States, 2016

Program (Blinded Name)	Health Care Organization	Program Partners	Target Patients	Chronic Disease Component
A	Nonprofit community health system	Community partner organization with patient navigators	Adults and families of children aged 18 years or younger who screen positive	None
B	Seven nonprofit hospitals and health systems, including an academic health center, a children’s hospital, and an FQHC	Food bank	All patients aged 18 years or older	Diabetes
C	Nonprofit community hospital and health system	Community nonprofit organization, college of osteopathic medicine, city department of public health	All patients	Congestive heart failure, type 2 diabetes, obesity
D	County-run health system with FQHCs	Food bank, community nonprofit organization	Children and adults	None
E	Nonprofit integrated care delivery health system	Statewide nonprofit organization	Children and seniors	Diabetes
F	Eight hospitals and health systems, including nonprofit teaching hospitals, nonprofit community hospitals, and critical access hospitals, as well as FQHCs	Food network (farmers, policy makers, grocers), local health council, county health department, Head Start, social service agencies, senior centers, school health clinics	All community residents, pregnant women, children younger than 5 years, teenagers	Diabetes
G	Nonprofit health system	Regional food bank	All patients, seniors, teenagers	Obesity, diabetes, and other chronic diseases
H	Community safety-net clinics, including an academic health center and 3 county FQHCs	Nonprofit farm	All patients	Chronic diseases
I	Community FQHC, nonprofit health system, and hospital network	Food bank	All patients, seniors, infants	Diabetes
J	Nonprofit community critical-access hospital	Nonprofit senior services organization, local council on aging, community nonprofit organization for seniors	Seniors (aged ≥60 y)	None
K	State-operated mental health center, onsite FQHC	University psychiatry department, citywide farms partnership	Adult patients aged 18 years or older	Mental illness, diabetes, cardiovascular disease
L	Nonprofit academic health center medical school, FQHC, nonprofit residential substance abuse treatment facility	Nonprofit farm	All patients, youth, pregnant women	Diabetes, obesity, cancer, substance abuse
M	Academic health center, nonprofit safety-net hospital	Food bank, community nonprofit organization	All patients	None
N	Charitable nonprofit health care organization, acute-care nonprofit hospital	Food bank	All patients, with a focus on seniors	None
O	Nonprofit health system, medical and dental clinic affiliated with a public research university, FQHC, community mental health center, senior center	CSA farm cooperative, early childhood alliance, community early childhood development nonprofit organization	All patients, families that have young children (aged 0–8 y) in the home	Chronic disease, diabetes, prediabetes, obesity
P	FQHC with 8 clinics	Coalition of regional farmers markets	All patients, pregnant women	Type 1 diabetes, type 2 diabetes, hypertension, obesity
Q	Nonprofit health system, safety-net hospital	Food bank	All patients, seniors	None
R	Nonprofit integrated health delivery system, 13 hospitals, primary care practices	Local food initiative, regional food bank	All primary care patients	None
S	State university hospital, academic health center and affiliated medical clinics, nonprofit health system, nonprofit community dental services	Food bank	All patients, adults and their families	Chronic disease
T	Community nonprofit hospital, primary care departments	Community nonprofit organization, food bank, city hunger network	All patients, children, pregnant women	None
U	Nonprofit hospital	National nonprofit organization supporting a network of food banks	All patients, seniors	None
V	Medical school–affiliated health system, nonprofit academic health center	Community nonprofit organizations, food pantry	All patients, adults and children	Diabetes, obesity

Abbreviations: CSA, community supported agriculture; FQHC, federally qualified health center.

### Food insecurity screening

Nearly two-thirds of the programs (n = 14) conduct food insecurity screening using the Hunger Vital Signs screener questions: 1) “Within the past 12 months we worried whether our food would run out before we got money to buy more” and 2) “Within the past 12 months the food we bought just didn’t last and we didn’t have money to get more” (21). The remaining programs use screening questions developed specifically for the program or other metrics, such as income in relation to the federal poverty level. In all but one of the programs, patients are screened for food insecurity during the clinical encounter, which includes primary care clinics, prenatal visits, well child checkups, urgent care and emergency department visits, home-based medical care, hospital admissions or discharges, and specialized health care facilities (ie, for mental health, substance abuse, diabetes, congestive heart failure, or dentistry). Most programs (n = 18) screen patients of all ages for food insecurity; however, several programs indicated a focus on screening adults aged 50 years or older (n = 7) or children (n = 10) (in addition to including adults aged ≥50 years in screening). In most programs (n = 18), a positive screening result triggers referral to the program or other food resources in the community, although in some cases (n = 4) referrals are provided at the discretion of the health care professional.

Most commonly, programs routinely screen all patients (n = 14), and of those, many (n = 8) indicated that they integrate screening into the clinical workflow as a part of routine intake or examination procedures. However, several programs (n = 8) do not routinely screen all patients but instead conduct screening at the discretion of individual providers. More than half of the hospitals and health systems track food insecurity screening results in the patient’s electronic health record. Screenings are conducted by various people, including medical assistants, patient navigators, social workers, community health workers, administrative staff, home health care providers, dietitians, certified diabetes educators, nurses, physicians, and medical students serving as patient advocates.

### Food insecurity interventions

The stated goals of the programs include the following: reducing food insecurity (n = 17), increasing access to and consumption of healthy foods or fruits and vegetables (n = 13), reducing chronic disease and improving health outcomes (n = 9), providing education on healthy eating (n = 4), and reducing hospital readmissions (n = 2). Various strategies are used to achieve these goals. Of the 13 types of food insecurity interventions, the most commonly implemented intervention (n = 19) is a referral to or a list of food resources, including local food resources (eg, food banks, soup kitchens, fruit and vegetable vouchers) and federal benefit programs such as the Supplemental Nutrition Assistance Program (SNAP) and the Special Supplemental Nutrition Program for Women, Infants, and Children (WIC) (Table 2). In 15 programs, a patient navigator, case manager, or social worker helps to connect patients with food insecurity to food resources, and in more than half of the programs (n = 14) they assist food-insecure patients in applying for federal benefits like SNAP, WIC, Medicaid, and Medicare. Most programs (n = 13) have an education component. Seven programs address a patient’s immediate need for food by hosting an onsite food pharmacy (n = 2) or food pantry (n = 5) in the health care setting. Seven programs provide medically tailored food, for example, food that is appropriate for helping patients manage their diabetes. Eight programs, commonly called fruit and vegetable prescription programs, provide vouchers or coupons for fresh fruits and vegetables that can be redeemed at farmers markets or food pantries. The most common combination of interventions, implemented concurrently by half of the programs (n = 11), is to provide a referral to or list of food resources, a patient navigator or case manager, and assistance with applications for federal benefits.

**Table 2:** Food Insecurity Intervention Types and Frequency of Implementation, 22 Health Care Entity–Based Programs, Tackling Hunger Landscape Assessment, United States, 2016

Intervention Type	Description	Number of Programs Implementing
Referral to outside resources	Patient is referred to or provided with a list of local or federal food resources (eg, referred to local food bank)	19
Patient navigator	Patient navigator, case manager, or social worker connects the patient to food resources	15
Federal benefit application assistance	Patient assisted in applying for federal benefits, either through a patient navigator or other case worker	14
Patient education	Patient provided with group classes or individual counseling on cooking, gardening, nutrition, or disease self-management	13
Fruit and vegetable vouchers	Patient provided vouchers or coupons for fresh fruits and vegetables that can be redeemed at farmers markets/food pantries (eg, a fruit and vegetable prescription program)	8
Medically tailored food	Patient provided medically tailored food (eg, tailored to patients with diabetes), either through home delivery or patients pick up at the food pantry	7
Onsite food pantry	Hospital or health care entity has an onsite food pantry that provides patients with emergency food	5
Boxes of healthy food, fresh produce, or both	Patient provided healthy food boxes, fresh fruits and vegetables, or both	4
Subsidized CSA shares	Patient given subsidized CSA shares to obtain fresh fruits and vegetables	3
Onsite food pharmacy	Hospital or health care entity has an onsite food pharmacy, which is similar to a food pantry but typically focuses on providing healthy foods, often through a prescription written by the health care provider	2
Onsite vegetable garden/farm or community garden	Patient provided produce from onsite vegetable garden/farm or community garden	2
SNAP matching	SNAP matching program that provides additional dollars when SNAP benefits are used at farmers markets	1
Subsidized healthy food for purchase	Patients provided healthy food for purchase at a subsidized (greatly reduced) price	1

Abbreviations: CSA, community supported agriculture; SNAP, Supplemental Nutrition Assistance Program.

### Funding and data collection

The programs receive long-term and short-term funds from various sources. More than half of the programs (n = 13) were informed by findings from a community health needs assessment (CHNA); consequently, common funding sources are the operating budgets of the health care entities (n = 11) and hospital community benefit funds (n = 9). Other common funding sources include foundations (n = 12), private donations (n = 5), USDA grants (n = 3), food banks (n = 8), and other city, state, and federal government funds (n = 7). Most programs (n = 14) indicated that they were financially sustainable for the next 2 to 3 years, while the remainder (n = 8) expressed concerns about long-term sustainability of funding.

Half of the programs (n = 11) have begun to track data on patient health outcomes, including body mass index, waist circumference, blood pressure, blood glucose, hemoglobin A1c, cholesterol, and hospital readmissions. Other data collected by programs include process indicators (number of patients enrolled in the program, referred to resources, and receiving food resources) and patient survey data on changes in food insecurity status, diet quality, fruit and vegetable intake, and knowledge of and self-efficacy around healthy eating.

## Discussion

This article describes 22 US health care entity–based programs that screen patients, including older adult patients, for food insecurity, and connect them with food resources. All but 3 of the programs were initiated after the enactment of the Affordable Care Act, which included changes in the tax code for tax years beginning after March 2012 that required nonprofit hospitals to conduct CHNAs. These changes influenced the community benefit investments made by nonprofit hospitals. Several key program features emerged from this review.

These programs were developed on the basis of strong partnerships between health care entities — often hospitals — and community-based organizations, with a focus on meeting needs identified in the community. Community partnerships are important, because partners may be abreast of the frequent changes in availability of food insecurity–related resources in the community that can meet the needs of both the patient and their household. More than half of the programs were informed by findings from a CHNA conducted by a nonprofit hospital that suggested that food insecurity was a key issue in the community. Several programs indicated that their food insecurity interventions were a part of a more holistic program that connects patients to resources for assistance with housing, transportation, utilities, education, vocational training, employment, child care, and English-language skills. A better understanding of the broader social needs of communities can help to target and tailor food insecurity interventions. Guidance for hospitals interested in assessing food systems and food security in their CHNAs, “Making Food Systems Part of Your Community Health Needs Assessment,” is available as a result of the Tackling Hunger project and accessible through the PHI (22). The guidance outlines key food insecurity indicators, data sources and measurement tools, and food security and food system stakeholders.

Screening is another important component of programs that leverage community–clinical linkages to address food insecurity. Most programs are using the Hunger Vital Signs screening questions (21) to screen patients for food insecurity. These 2 brief, easy-to-administer questions may be the most feasible screening tool for health care entity–based food insecurity programs. Research indicates that this tool provides a valid measure of food security status in various populations (23). Using the Hunger Vital Signs questions, the Hunger Safety Net Workgroup of the Nutrition and Obesity Policy Research and Evaluation Network has developed food insecurity screening algorithms to guide physicians in screening pediatric and senior patients for food insecurity and referring patients to emergency and long-term food resources in the community (24).

Many of the programs reviewed in this assessment have integrated screening into the workflow of the hospital or health care entity. Institutional policies to integrate screening may be important to ensure that screening is not conducted at the discretion of individual providers but is universal for all patients. Universal screening can be important to accurately identify and assist all patients with food insecurity. One way of integrating screening into the workflow is to track screening in electronic health records (EHRs), which is being done by more than half of the programs. A few programs have begun using software programs (25–27) to track referrals to and use of resources, with a desire to eventually assess impact on patient health outcomes. However, most of the programs are still in the early stages of such efforts, often attempting to use their EHR systems to link patient screening results with referrals, use of referred food resources, and health outcomes. This requires either 1) identifying one EHR software package that can track each of these program data elements as a patient moves through the program or 2) using applications that utilize technology based on FHIR (Fast Healthcare Interoperability Resources) and allow interoperability and exchange of patient data between multiple data collection systems. Moreover, considering that some programs aim to reduce hospital readmissions, identifying technology that is either programmed directly within the EHR or embedded within the EHR or other electronic data systems using FHIR-based resources will facilitate linkage of screening and referral data to readmission data at the patient level and will enable programs to determine their impact on health care use. A related challenge is complying with requirements to protect patient health information under the Health Insurance Portability and Accountability Act (HIPAA), including possible privacy issues when sharing patient referral data with program partners external to the health system.

The goal of many of the programs is to not only increase access to food in general but also improve access to healthy foods, including fresh fruits and vegetables, for chronic disease prevention. Some programs aim to support the local food system by increasing access to and consumption of fresh fruits and vegetables through farmers markets, subsidized community-supported agriculture shares, and food banks. These efforts are consistent with an increasing recognition among food banks that a large proportion of beneficiaries have chronic diseases and that food banks should provide nutritious foods that help prevent or manage chronic disease (28,29).

Many programs indicated that their funding was obtained from multiple short-term funding sources, and several program managers expressed concern about long-term sustainability. The sustainability of certain hospital-based food insecurity programs may depend on identifying secure funding, one example of which may be the USDA Food Insecurity Nutrition Incentive (FINI) Grant Program (30). FINI grants support projects that incentivize the purchase of fruits and vegetables among low-income consumers participating in SNAP and can help fund fruit and vegetable prescription programs and initiatives that provide SNAP matching funds at points of sale such as farmers markets.

Another potential source of funding could be support from public or private health insurance. Information that demonstrates that food security interventions can have a positive impact on patient health outcomes and reduce health care utilization will likely be important to this group. Although half of the programs have begun to track data on patient health outcomes, the other half have focused their data collection on measuring program enrollment, referrals to and use of resources, and changes in food insecurity status and diet quality. This focus could expand to include health outcomes and health care utilization to ensure program value and sustainability.

A particular area of interest in this assessment was describing health care entity–based food security programs that focus on older adults, and we found that nearly one-third of the programs we assessed had this focus. Food insecurity in older adults can have causes and consequences that are unique to this population. Seniors often have low levels of participation in food assistance programs — nearly 3 in 5 seniors who qualify for SNAP do not participate (31). Strong, sustainable clinical–community partnerships are needed to ensure that older adults who screen positive for food insecurity are referred to and participate in food assistance programs. Community and clinical interventions should be designed to reflect the unique needs and determinants of food insecurity in older patients and to address their barriers to participation in food assistance programs. Existing models for home and community-based nutrition and aging services can be expanded and combined with efforts to increase awareness of the needs of older adults.

To our knowledge, this is the first assessment of its kind to describe the range of health care entity–based programs in the United States that screen patients for food insecurity and connect them with food resources. A key strength of this assessment was that programs were identified by nominations through an extensive, national network of researchers, public health professionals, and health care entities involved in food security initiatives, and there was diverse geographic representation among the programs. Additionally, detailed information was gathered from programs in 3 separate steps using different methods — an online nomination form, formal document collection, and telephone interviews — which allowed for an in-depth assessment of key features of these programs.

However, this assessment has several limitations. First, the programs represent a convenience sample and were not a complete census of programs that meet the inclusion criteria. Additionally, the inclusion criteria narrowed the scope of programs that were reviewed, and useful approaches for screening patients for food insecurity and linking them to food resources may not have been captured in this assessment if formal screening was not conducted or the target population did not include older adults. The nomination process identified several established programs that screen for food insecurity only among children, during well child checkups or in pediatric hospitals, but these programs were not included in this assessment because the intervention excluded older adults. Future research could include a broader landscape assessment that identifies programs that focus only on children or programs that address food needs through a more general social needs assessment but that do so without conducting formal screening for food insecurity. For example, programs that use community health workers or case managers may address a patient’s food needs through a more comprehensive evaluation of social needs, and this type of program may represent a larger share of health care–linked food insecurity interventions in the United States. Lastly, this article describes health care entity–based food insecurity programs that were identified through a larger initiative to assess the readiness of these programs for a formal evaluation. It is important to note that these 22 programs have not yet undergone formal evaluation, and most had collected minimal data on effectiveness. Therefore, program effectiveness and best practices cannot be determined from this assessment.

Although many of the programs featured in this assessment are still in the early stages of implementation and are operating on a small scale with a limited reach, collectively they represent the momentum in the health care sector to focus on prevention and address the health impact of food insecurity in their communities. More evidence is needed about the effectiveness of health care entity–based programs in improving food security and health outcomes and decreasing health care utilization. Rigorous effectiveness research can provide information to potential stakeholders, including health care entities, community organizations, and public and private insurers, to guide their investments.
